# The Effect of Health-Related Behaviors on Disease Progression and Mortality in Early Stages of Chronic Kidney Disease: A Korean Nationwide Population-Based Study

**DOI:** 10.3390/jcm8081100

**Published:** 2019-07-25

**Authors:** Yookyung Lee, SuYeon Kwon, Jong Joo Moon, Kyungdo Han, Nam-Jong Paik, Won-Seok Kim

**Affiliations:** 1 Department of Rehabilitation Medicine, Seoul National University College of Medicine, Seoul National University Bundang Hospital, Seongnam-si, Gyeonggi-do 13620, Korea; 2 Department of Internal Medicine, Seoul National University Hospital, Seoul 03080, Korea; 3 Department of Biostatistics, The Catholic University of Korea College of Medicine, Seoul 06591, Korea

**Keywords:** chronic kidney disease, disease progression, end stage renal disease, mortality, health-related behaviors, physical activity, smoking, alcohol

## Abstract

A healthy life style is associated with decreased risk of chronic kidney disease (CKD) and mortality in the general population. However, there is no definitive evidence of the benefits of physical activity and other health-related behaviors in the early-stage of CKD. This study aimed to explore the association between health-related behaviors and end-stage renal disease (ESRD) and mortality in the early stages of CKD. The National Health Insurance Service (NHIS) database from 1 January 2009 to 31 December 2016 was used to screen 83,470 subjects with early stage CKD. Cox proportional hazard regression analysis was used to evaluate the association between health-related behaviors and ESRD and death. Kaplan–Meier curves for mortality and ESRD were plotted according to the physical activity, smoking status, and alcohol consumption pattern. Risk of death decreased significantly in subjects who engaged in sufficient physical activity (adjusted Hazard Ratio (HR) 0.73; 95% CI: 0.64–0.83; *p* < 0.001). Risk of ESRD and death increased significantly in the current smoker with adjusted HR of 1.44 (95% CI: 1.06–1.95; *p* < 0.02) and 1.61 (95% CI: 1.44–1.80; *p* < 0.001) respectively. Therefore, systematic interventions to encourage physical activity and smoking cessation need to be actively considered in the early stages of CKD.

## 1. Introduction

Chronic kidney disease (CKD) is a major global health burden due to its high prevalence and economic cost. The worldwide prevalence of CKD is estimated to be from 11% to 13% [1]. Increasing prevalence of hypertension, diabetes, and aging in societies suggests that the number of CKD patients will further increase in the future [2]. In developed countries, more than 1% of the total health budget is dedicated to the treatment of 0.1% of the population with end-stage renal disease (ESRD) [3].

Previous studies have investigated the relationship between health-related behaviors and CKD in the general population [4,5,6,7]. Regular exercise was associated with lower risk of CKD [7]. In a recent meta-analysis, smoking was shown to be an independent risk factor for CKD [6]. Alcohol consumption was associated with decreased risk of CKD [4]. These studies imply that lifestyle modification may be important in preventing CKD.

According to the 2012 Kidney Disease Improving Global Outcomes (KDIGO) clinical practice guidelines, exercise and smoking cessation are recommended for CKD patients [8]. Exercise may delay the decline of kidney function [9] and lower the risk of cardiovascular disease (CVD), for which CKD is a risk factor [10]. Although the benefits of healthy life style on disease progression and mortality in CKD patients seem straightforward, evidence is sparse, especially in patients with early stages of CKD, who may be the best candidates to benefit from health behavior modifications. Most studies evaluating the relationship between exercise and CKD have been limited by small sample size [9]. Studies including early-stage CKD patients are relatively few and inconclusive [11]. There have been mixed results with respect to the effect of alcohol on CKD, with some reporting protective effects while other reporting increased risk [12,13].

Therefore, the objective of this study was to investigate the real-world impact of health-related behavioral change on CKD progression and mortality using nationally representative data.

## 2. Materials and Methods

### 2.1. Data Source

Most Koreans (97.0%) residing in Korea are covered by one of two health care programs under the National Health Insurance system: The National Health Insurance (NHI) or Medical Aid (MA) [14]. Information on patient demographics, medical service use, disease diagnosis, and life style from the two health care programs is incorporated into a single National Health Insurance Service (NHIS) database accessible for researchers. The Korean NHIS also provides biannual health check-ups which include a questionnaire on past medical history and health-related behaviors, measurements of height, weight, blood pressure, blood count, and blood chemistry test [14].

### 2.2. Study Cohort for Early Stages of CKD

**Figure 1:**
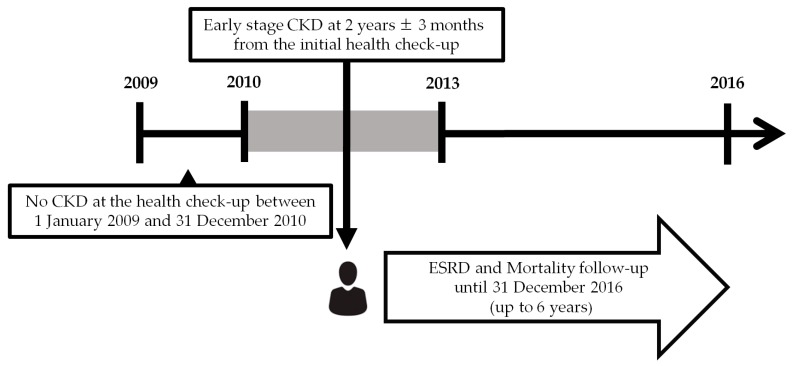
Study design.

Using the Korean NHIS database (NHIS-2019-1-101), subjects who received regular health check-up between 1 January 2009 and 31 December 2010 were screened (Figure 1). The inclusion criteria were as follows: (1) age 20 to 80 years; (2) without CKD as defined by the estimated glomerular filtration rate (eGFR) ≥90 mL/min/1.73 m^2^ and negative urine dipstick test at the initial health check-up; (3) record of follow-up health check-up at two years ± three months from the initial health check-up; and (4) fit the CKD diagnostic criteria, as defined by eGFR >60 mL/min/1.73 m^2^, and a positive urine dipstick test or eGFR between 30 to 59 mL/min/1.73 m^2^ at the follow-up health check-up (index year) [8]. Subjects with missing data on variables included in the statistical analysis or already diagnosed as ESRD at index year were excluded.

### 2.3. Health-Related Behaviors

Health-related behaviors were assessed based on the self-reported questionnaire included in the regular health check-up. The physical activity questionnaire consisted of 3 questions on physical activities performed during the last 7 days (Supplementary Material). Sufficient physical activity was defined as (1) 20 min or more of vigorous physical activity such as running, aerobics, fast bicycling, or mountain climbing performed at least 3 days a week; or (2) 30 min or more of moderate physical activity, such as fast walking, doubles tennis, or bicycling at a regular pace, performed at least 5 days a week; or (3) 4 days of moderate and 1 to 2 days of vigorous physical activity; or (4) 3 days of moderate and 2 days of vigorous physical activity, based on the International Physical Activity Questionnaire (IPAQ) scoring protocol of moderate or high levels of physical activity [15,16]. A current smoker was defined as having smoked more than 100 cigarettes in a life time and who was currently smoking daily or intermittently, according to the Centers for Disease Control and Prevention tobacco glossary [17]. Alcohol consumption was divided into three levels: Heavy drinker, defined as drinking more than 30 g alcohol per day; mild drinker, defined as less than 30 g alcohol per day; and nondrinker, defined as drinking no alcohol at all [18,19].

### 2.4. ESRD and Mortality

Subjects were followed-up until 31st December 2016 for the outcome event of ESRD or mortality. ESRD was defined as the relevant International Classification of Diseases, 10th revision, Clinical Modification (ICD-10-CM) codes (N18-19, Z49, Z94.0, Z99.2) combined with the initiation of renal replacement therapy (R3280, O7011-O7020 or V001, O7071-O7075 or V003). Death from any cause was also obtained from the Korean NHIS database.

### 2.5. Covariate Data

Demographic data, such as age, sex, area of residence, and income level were collected. Urban residence was grossly defined as an area with a population of greater than 50,000 according to the Korean Local Autonomy Act. Subjects in the lowest quantile in national health insurance payment or recipients of medical aid were grouped into the low income group.

Past medical history, such as diabetes mellitus, hypertension, dyslipidemia, and a history of CVD was also gathered. Diabetes mellitus was defined by fasting glucose ≥126 mg/dL or ICD-10-CM codes of E11-E14 and at least one claim per year for the prescription of antidiabetic medication. Hypertension was defined by blood pressure (BP) ≥140/90 or disease codes of I10-I13, and I15, and at least one claim per year for the prescription of antihypertensive medication. Dyslipidemia was defined by total cholesterol ≥240 mg/dL or a code of E78 and at least one claim per year for the prescription of lipid lowering medication. History of CVD was determined by self-report. If subjects answered yes to one of the following two questions, they were considered to have a history of CVD: (1) Have you been diagnosed or are currently on medication for stroke? (2) Have you been diagnosed or are currently on medication for heart disease (myocardial infarction/angina)?

Charlson Comorbidity Index (CCI) was acquired using ICD-10-CM diagnoses of patients [20]. Comorbid conditions were assigned weighted scores based on the relative risk of one year death from the comorbid condition and summed to yield the CCI score. CCI was categorized into three groups: 0, 1, and ≥2.

Blood chemistry test results, such as eGFR (calculated using the Modification of Diet in Renal Disease (MDRD) equation), fasting plasma glucose level, serum low-density lipoprotein (LDL), and urine dipstick, body mass index (BMI), and blood pressure, were collected. Fasting glucose, LDL, BMI, and blood pressure were categorized according to the recommended target values in CKD patients (Fasting glucose 90–130 mg/dL, LDL 70–100 mg/dL, BMI 20–25 kg/m^2^, BP < 130/80 mmHg) [8]. Urine dipstick results were categorized as negative, trace, and 1+ or more.

### 2.6. Statistical Analysis

The baseline characteristics were presented as the mean ± standard deviation or as a number with percentage. Chi-square test was applied for categorical variables, and Student’s *t* test was applied for continuous variables to compare the characteristics between the ‘Death’ and ‘No Death’ groups, as well as between the ‘ESRD’ and ‘No ESRD’ groups. Cox proportional hazard regression analysis was applied to evaluate the association between health-related behaviors (physical activity, smoking, and alcohol) and ESRD and death. The results were adjusted for age, sex, area of residence, income, diabetes mellitus, hypertension, dyslipidemia, history of CVD, health-related behaviors, categorized CCI, urine dipstick, fasting glucose, LDL, BMI, and BP. Kaplan–Meier curves for mortality and ESRD were plotted according to the physical activity, smoking, and alcohol. The log rank test was performed to analyze the group differences. All statistical analyses were performed using SAS 9.4 (SAS Institute Inc., Cary, NC, USA). A two sided *p* value < 0.05 was considered statistically significant.

## 3. Results

### 3.1. Baseline Characteristics

**Figure 2:**
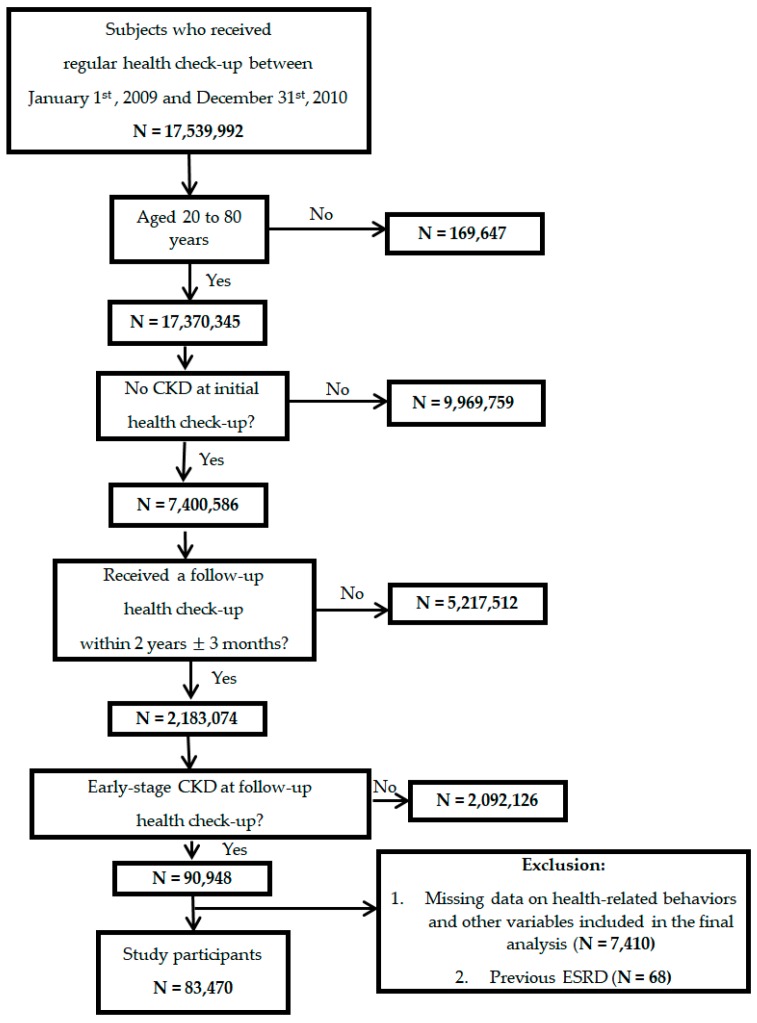
Patient flow. CKD, Chronic Kidney Disease; ESRD, End Stage Renal Disease.

A total of 83,470 subjects with early stages of CKD (mean age 48.43 years; mean baseline eGFR 90.48 mL/min/1.73 m^2^; male 54%) were included in this study (Figure 2). Approximately 16% of the subjects had diabetes mellitus, 33% had hypertension, 26% had dyslipidemia, and 2% had a history of CVD. Around 25% of the subjects had a CCI score of 2 or more. The number of subjects who met the target blood pressure of less than 130/80 mmHg, fasting glucose of 90 to 130 mg/dL, LDL of 70 to 100 mg/dL, and BMI of 20 to 25 kg/m^2^ was 38,825 (46.51%), 46,947 (56.24%), 22,478 (26.93%), and 43,758 (52.42%) respectively (Table 1). Statistically significant differences were noted between the ‘ESRD’ and ‘No ESRD groups’, and ‘Death’ and ‘No Death’ groups in age, sex, area of residence, income levels, past medical history of diabetes mellitus, hypertension, dyslipidemia, history of CVD, CCI, smoking status, alcohol consumption levels, eGFR, urine dipstick results, fasting plasma glucose, and blood pressure levels. LDL and BMI levels were significantly different between the ‘Death’ and ‘No Death’ groups but not the ‘ESRD’ and ‘No ESRD’ groups (Table 1).

### 3.2. Health-Related Behaviors and Risk of ESRD and Death

#### 3.2.1. Physical Activity

**Figure 3:**
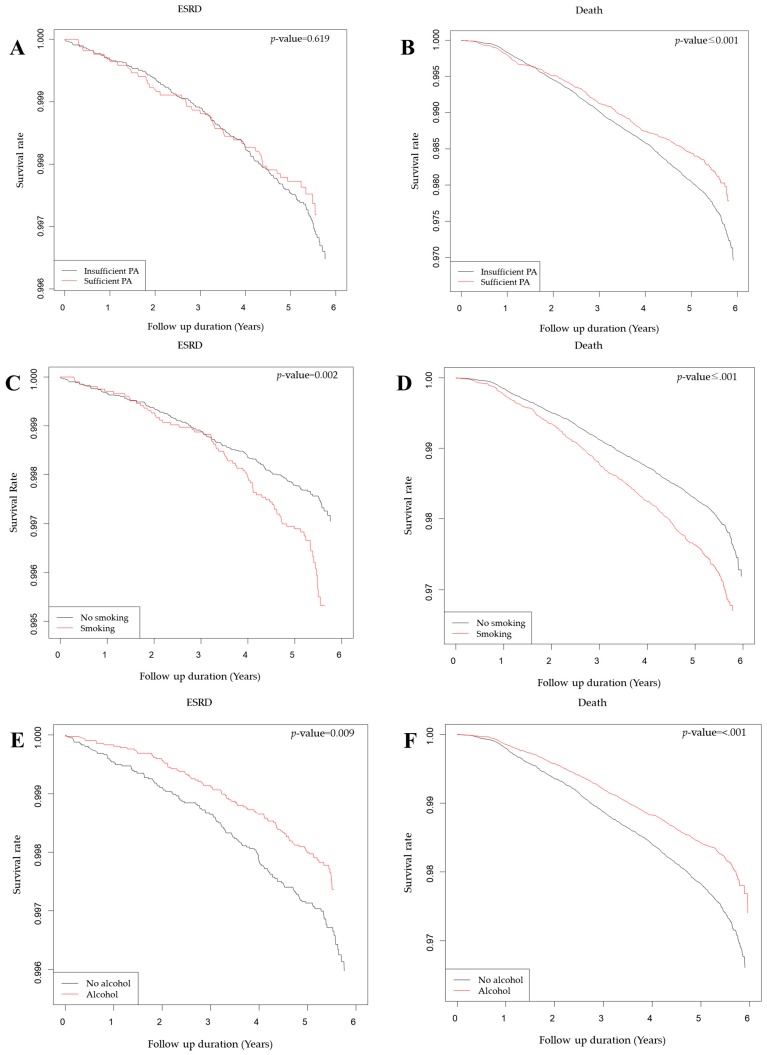
Kaplan–Meier curves for end-stage renal disease (ESRD) and mortality according to sufficient physical activity (PA) (**A**,**B**), smoking (**C**,**D**), and alcohol consumption (**E**,**F**).

Approximately 20% of subjects reported to be engaged in sufficient physical activity. There was a significantly higher survival rate in the sufficient physical activity group, but no significant difference in the ESRD progression rate in the unadjusted Kaplan–Meier analysis (Figure 3A,B). The risk of death was significantly decreased in subjects who engaged in sufficient physical activity (adjusted Hazard Ratio (HR) 0.73; 95% CI: 0.64–0.83; *p* < 0.001) (Table 2). In addition, a tendency towards decreased risk of ESRD was observed, but without statistical significance (adjusted HR 0.84; 95% CI: 0.60–1.17; *p* = 0.30).

#### 3.2.2. Smoking

Approximately 25% of the total subjects were current smokers. According to the unadjusted Kaplan–Meier analysis, current smokers showed a significantly lower survival rate and higher ESRD progression rate (Figure 3C,D). Risk of ESRD and death increased significantly in the current smoking group with respective adjusted HR of 1.44 (95% CI: 1.06–1.95; *p* < 0.02), and 1.61 (95% CI: 1.44–1.80; *p* < 0.001) (Table 2).

#### 3.2.3. Alcohol Consumption

Approximately 50% of total subjects were alcohol drinkers. Alcohol drinkers showed a significantly higher survival rate and lower ESRD progression rate in the unadjusted Kaplan–Meier analysis (Figure 3E,F). The risk of ESRD and death decreased significantly in the alcohol drinking group with adjusted HR of 0.59 (95% CI: 0.44–0.79; *p* < 0.001) and 0.83 (95% CI: 0.74–0.93; *p* < 0.001), respectively (Table 2).

## 4. Discussion

Health-related behaviors are associated with disease progression and mortality in CKD patients. Subjects who engage in sufficient physical activity had a significantly lower risk of death than those with insufficient physical activity. Current smokers had a significantly higher risk of ESRD and death than their non-smoking counterparts. Alcohol drinkers had a significantly lower risk of ESRD and death than non-drinkers (Table 2).

A prospective cohort study, based on the Cardiovascular Health Study, reported that physical activity decreased the risk of CKD progression, as defined by a loss of 3.0 mL/min/1.73 m^2^ per year in GFR [21]. In our study, the risk of CKD progression, as defined by ESRD, was not significantly lower in subjects who engage in sufficient physical activity. This may have been because ESRD as an endpoint was less sensitive than the decrease in GFR by certain percent or amount. In addition, the follow-up time of up to 6 years may have been short in detecting the occurrence of ESRD in our study of subjects with early-stage CKD. A previous study showed that patients had minimal progression of CKD over a period of 6 years [22].

Previous studies reported that physical activity decreased all-cause mortality in the general population and in ESRD patients on renal replacement therapy [23,24,25]. Our study reproduced the results of previous studies in the early-stage CKD population. Although the exact mechanism remains uncertain, exercise is thought to decrease the risk of death by improving metabolic factors and decreasing risk of CVD [26]. In addition, physical activity can reduce muscle wasting, which is a cause of premature death in ESRD patients [27,28]. Despite benefits of physical activity in CKD patients, only 20% of patients engaged in sufficient physical activity (Table 1). Therefore, more aggressive life style interventions and systematic efforts are needed in patients in the early-stage CKD to delay disease progression and possible death.

Smoking significantly increased the risk of ESRD and death in early-stage CKD patients. Our study reproduced the results of previous studies. A recent meta-analysis study showed that current smokers had an OR of 1.91 (95% CI: 1.39–2.64) for ESRD [6]. Mortality HR for all causes in male and female current smokers with CKD were 2.26 (95% CI: 1.95–2.63) and 1.78 (95% CI: 1.36–2.32), respectively [29]. Despite the detrimental effects of smoking on CKD progression and death in CKD patients, 25% of patients with early-stage CKD were still currently smoking (Table 1). Therefore, active interventions to quit smoking are also needed to be incorporated in routine health-checkups with a focus on patients with early-stage CKD.

Alcohol consumption was associated with lower risk of ESRD and death in patients with early-stage CKD. The results of previous studies on the effect of alcohol consumption and risk of ESRD and death in CKD patients have been mixed. Contrary to our results, a previous meta-analysis reported a relative risk (RR) of 1.00 (95% CI: 0.55–1.82) for ESRD in CKD patients with high alcohol consumption [5]. This may have been due to the differences in the method of comparison of alcohol consumption. Our study compared no alcohol consumption with alcohol consumption, while the meta-analysis compared alcohol consumption with high alcohol consumption. A previous study reported a mortality risk for alcohol consumption of more than 2 drinks a week in CKD patients as 0.67 (95% CI: 0.48–0.94) [10]. Alcohol is thought to prevent hyalinization of renal arterioles, increase high-density lipoprotein (HDL) cholesterol, and reduce inflammation by controlling the plasma fibrinogen and IL-1 alpha levels [4]. Polyphenol in red wine has an antioxidant effect. These mechanisms are thought to be involved in the protection of kidney function with alcohol consumption [4]. However, caution is needed in interpreting these results into clinical practice, since the optimal alcohol consumption level is not yet clear and may be hard to control.

The strengths of our study are as follows: Large sample size of patients in the early stages of CKD, nationally representative data, and longitudinal follow-up of up to 6 years. Detailed information from the health-checkup data was used, and possible confounders such as socioeconomic variables, comorbidities, and blood chemistry test results were included into a comprehensive analysis.

Nonetheless, there are several limitations to consider as well. First, urine dipstick was used in the definition of CKD instead of proteinuria, which is more sensitive. Moreover, assessment of proteinuria based on a single measurement of urine dipstick may be unreliable due to high variability of low-degree proteinuria [30]. Second, the data on health-related behaviors were based on self-report, which may be highly subjective and not very reliable. Studies have shown that subjects tend to overestimate their physical activity level in self-reports [31]. However, if physical activity levels were overestimated, it only implies that more subjects do not engage in sufficient physical activity and that mortality risk can be decreased with even less physical activity. Similarly subjects tend to underreport alcohol consumption [5]. Since our study compared the no-alcohol consumption group to the alcohol consumption group, slight underestimation of alcohol consumption will likely not impact our study results. Third, subjects who did not receive a health check-up were excluded, increasing the risk of selection bias. Fourth, not all possible confounding factors, such as medication, sarcopenia, nutritional status, or dietary intake, were considered [32]. Fifth, dyslipidemia was defined by total cholesterol ≥240 mg/dL and not by LDL cholesterol measurement, which is clinically more significant as a risk factor for CVD [33]. Lastly, the different etiologies of CKD of various age groups, which could affect the prognosis, were not taken into consideration.

## 5. Conclusions

In early CKD patients, sufficient physical activity is associated with decreased risk of mortality. Smoking is associated with increased risk of ESRD and death. However, only 20% of CKD patients engaged in sufficient physical activity and 25% of patients continued to smoke. Therefore, interventions to modify health-related behaviors during the early stages of CKD should be considered and promoted.

## Figures and Tables

**Table 1:** Baseline characteristics of study population.

*n*	Total	Death			ESRD		
		No	Yes	*p* Value	No	Yes	*p* Value
	83,470	81,664	1,806		83,237	233	
Age, years, mean ± SD	48.43 ± 13.34	48.09 ± 13.16	64.05 ± 12.02	<0.001	48.41 ± 13.33	57.24 ± 12.97	<0.001
Male, *n*, (%)	45,108(54.04)	43,835(53.68)	1,273(70.49)	<0.001	44,935(53.98)	173(74.25)	<0.001
Urban residence, *n*, (%)	37,732(45.2)	37,064(45.39)	668(36.99)	<0.001	37,652(45.23)	80(34.33)	<0.001
Low Income, *n*, (%)	12,956(15.52)	12,562(15.38)	394(21.82)	<0.001	12,893(15.49)	63(27.04)	<0.001
Diabetes Mellitus, *n*, (%)	13,047(15.63)	12,383(15.16)	664(36.77)	<0.001	12,931(15.54)	116(49.79)	<0.001
Hypertension, *n*, (%)	27,346(32.76)	26,275(32.17)	1,071(59.3)	<0.001	27,188(32.66)	158(67.81)	<0.001
Dyslipidemia, *n*, (%)	21,377(25.61)	20,801(25.47)	576(31.89)	<0.001	21,275(25.56)	102(43.78)	<0.001
History of CVD, *n*, (%)	1,326(1.59)	1,198(1.47)	128(7.09)	<0.001	1,312(1.58)	14(6.01)	<0.001
CCI, *n*, (%)				<0.001			<0.001
0	42,039(50.36)	41,654(51.01)	385(21.32)		41,994(50.45)	45(19.31)	
1	20,782(24.9)	20,452(25.04)	330(18.27)		20,751(24.93)	31(13.3)	
2 or more	20,649(24.74)	19,558(23.95)	1,091(60.41)		20,492(24.62)	157(67.38)	
Sufficient physical activity, *n*, (%)	16,854(20.19)	16,550(20.27)	304(16.83)	<0.001	16,810(20.2)	44(18.88)	0.619
Smoking, *n*, (%)				<0.001			<0.001
No	49,476(59.27)	48,590(59.5)	886(49.06)		49,367(59.31)	109(46.78)	
Ex	13,451(16.11)	13,086(16.02)	365(20.21)		13,405(16.1)	46(19.74)	
Current	20,543(24.61)	19,988(24.48)	555(30.73)		20,465(24.59)	78(33.48)	
Alcohol consumption, *n*, (%)				<0.001			0.007
No	41,602(49.84)	40,533(49.63)	1,069(59.19)		41,466(49.82)	136(58.37)	
Mild	34,622(41.48)	34,102(41.76)	520(28.79)		34,549(41.51)	73(31.33)	
Heavy	7,246(8.68)	7,029(8.61)	217(12.02)		7,222(8.68)	24(10.3)	
eGFR, ml/min/1.73 m^2^, *n*, (%)				<0.001			<0.001
30–59	16,365(19.61)	15,760(19.3)	605(33.5)		16,269(19.55)	96(41.2)	
60–89	25,042(30)	24,508(30.01)	534(29.57)		24,963(29.99)	79(33.91)	
90-	42,063(50.39)	41,396(50.69)	667(36.93)		42,005(50.46)	58(24.89)	
Urine Dipstick, *n*, (%)				<0.001			<0.001
Negative	15,344(18.38)	14,820(18.15)	524(29.01)		15,284(18.36)	60(25.75)	
Trace	39,384(47.18)	38,822(47.54)	562(31.12)		39,341(47.26)	43(18.45)	
1+ or more	28,742(34.43)	28,022(34.31)	720(39.87)		28,612(34.37)	130(55.79)	
Fasting Glucose, *n*, (%)				<0.001			<0.001
<90	27,850(33.37)	27,508(33.68)	342(18.94)		27,798(33.4)	52(22.32)	
90–130	46,947(56.24)	45,909(56.22)	1,038(57.48)		46,837(56.27)	110(47.21)	
>130	8,673(10.39)	8,247(10.1)	426(23.59)		8,602(10.33)	71(30.47)	
LDL, mg/dL, *n*, (%)				<0.001			0.059
<70	7,924(9.49)	7,596(9.3)	328(18.16)		7,894(9.48)	30(12.88)	
70–100	22,478(26.93)	21,992(26.93)	486(26.91)		22,407(26.92)	71(30.47)	
>100	53068(63.58)	52076(63.77)	992(54.93)		52,936(63.6)	132(56.65)	
BMI, *n*, (%)				<0.001			0.631
<20	10,247(12.28)	9,960(12.2)	287(15.89)		10,223(12.28)	24(10.3)	
20–25	43,758(52.42)	42,774(52.38)	984(54.49)		43,635(52.42)	123(52.79)	
>25	29,465(35.3)	28,930(35.43)	535(29.62)		27,379(35.3)	86(36.91)	
BP > 130/80 mmHg, *n*, (%)	44,645(53.49)	43,452(53.21)	1,193(66.06)	<0.001	44,481(53.44)	164(70.39)	<0.001

Statistical analysis: Chi-square test and Student’s *t* test. Abbreviations: SD, standard deviation; CVD, cardiovascular disease; CCI, Charlson comorbidity index; eGFR, estimated glomerular filtration rate; LDL, low density lipoprotein; BMI, body mass index; BP, blood pressure.

**Table 2:** Health-related behaviors and risk of ESRD and death.

	N	ESRD	Follow-up Duration (Person × Year)	Crude HR (95%CI)	Adjusted HR (95%CI) ^a^	Death	Follow-up Duration (Person × Year)	Crude HR (95%CI)	Adjusted HR (95%CI) ^a^
**Smoking**									
Non, Ex	62,927	155	336,424.23	1(ref.)	1(ref.)	1,251	336,675.06	1(ref.)	1(ref.)
Current	20,543	78	108,623.67	1.41 (1.04,1.90)	1.44 (1.06,1.95)	555	108,733.86	1.7 (1.52,1.90)	1.61 (1.44,1.80)
*p*-value				0.03	0.02			<0.001	<0.001
**Alcohol**									
Non	41,602	136	222,490.34	1(ref.)	1(ref.)	1,069	222,719.88	1(ref.)	1(ref.)
<30 g/day or >30 g/day	41,868	97	222,557.55	0.60 (0.45,0.80)	0.59 (0.44,0.79)	737	222,689.04	0.87 (0.79,0.97)	0.83 (0.74,0.93)
*p*-value				<0.001	<0.001			0.01	<0.001
**Physical Activity**									
Insufficient	66,616	189	354,888.68	1(ref.)	1(ref.)	1,502	355,182.57	1(ref.)	1(ref.)
Sufficient	16,854	44	90,159.22	0.79 (0.57,1.1)	0.84 (0.60,1.17)	304	90,226.35	0.69 (0.61,0.78)	0.73 (0.64,0.83)
*p*-value				0.16	0.30			<0.001	<0.001

^a^ Adjusted for age, sex, area of residence, income, diabetes mellitus, hypertension, dyslipidemia, history of CVD, CCI, exercise, smoking, alcohol, urine dipstick, fasting glucose, LDL, BMI, BP. Statistical analysis: Cox proportional hazard regression analysis. Abbreviations: ESRD, end stage renal disease; HR, hazard ratio; 95% CI, 95% confidence interval; CVD, cardiovascular disease; CCI, Charlson comorbidity index; LDL, low density lipoprotein; BMI, body mass index; BP, blood pressure.

## Supplementary Materials

The materials are available online at https://www.mdpi.com/2077-0383/8/8/1100/s1.
