# Supplementary material for: The Effect of Health-Related Behaviors on Disease Progression and Mortality in Early Stages of Chronic Kidney Disease: A Korean Nationwide Population-Based Study
Source: J Clin Med. 2019 Jul 25;8(8):1100. doi: 10.3390/jcm8081100 (PMC6723181; doi:10.3390/jcm8081100)
Supplement: Supplementary file 1 [file jcm-08-01100-s001.pdf]

Supplementary Material: Translation of the physical activity questionnaire

Please read the following questions and check the answer which corresponds to your physical activity status of the past week.

1. During the past week, how many days did you engage, for more than 20 minutes, in vigorous physical activities which made you notably more short of breath than usual? (e.g.: running, aerobics, fast bicycling or mountain climbing)

   ☐ 0 ☐ 1 ☐ 2 ☐ 3 ☐ 4 ☐ 5 ☐ 6 ☐ 7

2. During the past week, how many days did you engage, for more than 30 minutes, in moderate physical activities which made you a little more short of breath than usual? (e.g.: fast walking, doubles tennis or bicycling at a regular pace)

   ☐ 0 ☐ 1 ☐ 2 ☐ 3 ☐ 4 ☐ 5 ☐ 6 ☐ 7

3. During the past week, how many days did you walk at least 10 minutes at a time for a total of more than 30 minutes a day? (e.g.: Light exercise, including walking to and from work and leisure time walking)

   ※ Exclude physical activities related to questions 1 and 2.

   ☐ 0 ☐ 1 ☐ 2 ☐ 3 ☐ 4 ☐ 5 ☐ 6 ☐ 7
